# Machine Learning Reveals Novel Pediatric Heart Failure Phenotypes with Distinct Mortality and Hospitalization Outcomes

**DOI:** 10.3390/diagnostics15222893

**Published:** 2025-11-14

**Authors:** Muhammad Junaid Akram, Asad Nawaz, Lingjuan Liu, Jinpeng Zhang, Haixin Huang, Bo Pan, Yuxing Yuan, Jie Tian

**Affiliations:** 1 Ministry of Education Key Laboratory of Child Development and Disorders, Department of Pediatric Cardiology, National Clinical Key Cardiovascular Specialty, National Clinical Research Center for Child Health and Disorders, Children’s Hospital of Chongqing Medical University, Chongqing 400014, China; drjunaidmalik2@gmail.com (M.J.A.); alic820@gmail.com (A.N.); liulingjuan_cool@163.com (L.L.); zjp1314615103@163.com (J.Z.); huanghaixin29@gmail.com (H.H.); bopan@hospital.cqmu.edu.cn (B.P.); 2 Key Laboratory of Children’s Important Organ Development and Diseases, Chongqing Municipal Health Commission, Chongqing 400014, China

**Keywords:** pediatric heart failure, machine learning, phenotyping, unsupervised clustering, cardiomyopathy, precision medicine

## Abstract

**Background**: Pediatric heart failure (PHF) is a heterogeneous syndrome with high morbidity, but existing classification systems inadequately capture its developmental and pathophysiological complexity due to reliance on adult-centric parameters. Using machine learning, we aimed to identify clinically distinct PHF phenotypes with unique outcomes and therapeutic implications. **Methods**: In this multicenter retrospective study, we analyzed 2903 consecutive PHF patients (≤18 years) from 30 Chinese tertiary centers from 20 provinces (2013–2022). Unsupervised machine learning (k-means clustering with PCA) evaluated 99 clinical, biomarker, and echocardiographic variables to derive phenotypes, which were compared for mortality, hospitalization, and treatment responses. **Results**: Three phenotypically distinct clusters emerged. Cluster 1 (Chronic Hypertensive and Cardiorenal Profile, 30.1%) predominantly affected older children (78%) with hypertension (54.4%), renal dysfunction (creatinine 45.8 μmol/L), and ventricular tachycardia (53.8%). This cluster showed the lowest in-hospital mortality (2.5%) but frequent 7–14 day hospitalizations (35.8%) and the highest beta-blocker use (54.5%). Cluster 2 (Preterm and CHD-Associated HF, 43.4%) comprised preterm infants (71.4%) with congenital heart disease (72.2%) and preserved LVEF (67%), demonstrating the highest mortality (5.1%) and prolonged stays (>30 days: 10.6%) with predominant diuretic (40.6%) and antibiotic use (54.3%). Cluster 3 (Fulminant Myocarditis Profile, 26.5%) exhibited cardiogenic shock with severely reduced LVEF (33%) and elevated BNP (3234 pg/mL), showing bimodal outcomes (4.8% LOS < 3 days vs. 32.2% LOS 15–30 days) and the highest IVIG utilization (46.5%) with intermediate mortality (3.8%). The majority of between-group differences were statistically significant (*p* < 0.001). **Conclusions**: Machine learning identified three PHF phenotypes with distinct in-hospital risk profiles and therapeutic implications, challenging current classification systems. These findings highlight the potential for phenotype-specific management strategies and provide a rationale for future research into arrhythmia prevention in hypertensive profiles and early immunomodulation in fulminant myocarditis, while highlighting the need for specialized care pathways for preterm/CHD patients. Prospective validation is warranted to translate this framework into clinical practice.

## 1. Introduction

Pediatric heart failure (PHF) is a devastating syndrome with heterogeneous etiologies, including congenital heart disease (CHD), cardiomyopathies, and myocarditis [1,2,3,4]. Despite advances in management, PHF carries substantial in-hospital mortality (7–26%) and hospitalization burdens, yet evidence-based strategies remain extrapolated from adult guidelines, which may be ill-suited to the unique aspects of pediatric pathophysiology [5,6,7,8,9]. This critical gap stems from fundamental limitations in current classification systems, which rely on reductionist parameters like left ventricular ejection fraction (LVEF) or symptomatic status—approaches that fail to capture three key pediatric-specific complexities. First, the dynamic interplay between developmental biology and disease mechanisms creates distinct clinical phenotypes across age strata. Second, conventional biomarkers (e.g., BNP) exhibit age-dependent variability that obscures true disease severity. Most critically, no framework exists to predict which children will abruptly decompensate versus stabilize, resulting in reactive rather than preemptive care [10,11,12,13,14,15,16].

Machine learning (ML) offers a transformative solution to this diagnostic impasse. In adult cardiology, unsupervised clustering has revealed phenogroups with differential treatment responses and outcomes [17,18,19]. However, pediatric applications remain scarce due to small single-center samples unable to capture PHF diversity and inappropriate borrowing of adult clustering variables that neglect developmental physiology [20,21,22]. To bridge this gap, we harnessed the largest multicenter PHF cohort (*n* = 2903) and applied unsupervised ML to 99 multidimensional variables—spanning clinical, biomarker, and imaging features. Our study sought to uncover latent PHF phenotypes purely data-driven from this heterogeneous population, dissect their distinct in-hospital mortality risks and hospitalization length, and decode real-world treatment patterns tied to each cluster. The resulting framework redefines PHF as a spectrum of biologically distinct disorders, challenging the current “one-size-fits-all” paradigm. By enabling early phenotype recognition for high-risk subgroups and providing a foundation for mechanistic studies, this work provides a foundation toward precision medicine in pediatric cardiology.

## 2. Methodology

### 2.1. Study Design and Data Collection

This multicenter retrospective cohort study was conducted across 30 tertiary medical centers in 20 Chinese provinces under the coordination of the National Center for Children’s Health Clinical Research (Table S1). We included pediatric patients (≤18 years) hospitalized with a primary diagnosis of heart failure between January 2013 and December 2022. Patient identification was performed through standardized hospital information systems to ensure consistency in case ascertainment. The study adhered to a predefined data collection protocol aligned with STROBE guidelines, building upon our previously validated methodology to maintain uniformity across participating centers [23]. This protocol included standardized variable definitions, data extraction procedures, and centralized data cleaning with quality control to minimize site-to-site variability and ensure data consistency.

### 2.2. Ethical Approval and Data Governance

Ethical approval was obtained from the Institutional Review Board of Chongqing Medical University (Approval No. 2020.160, Date of Approval: 15 April 2022), with a waiver of informed consent granted due to the retrospective nature of the study. All patient identifiers were scrubbed prior to database integration, ensuring confidentiality while preserving analytical integrity. The study complied with the Declaration of Helsinki principles and Chinese data protection regulations.

### 2.3. Study Population and Eligibility Criteria

**Figure 1:**
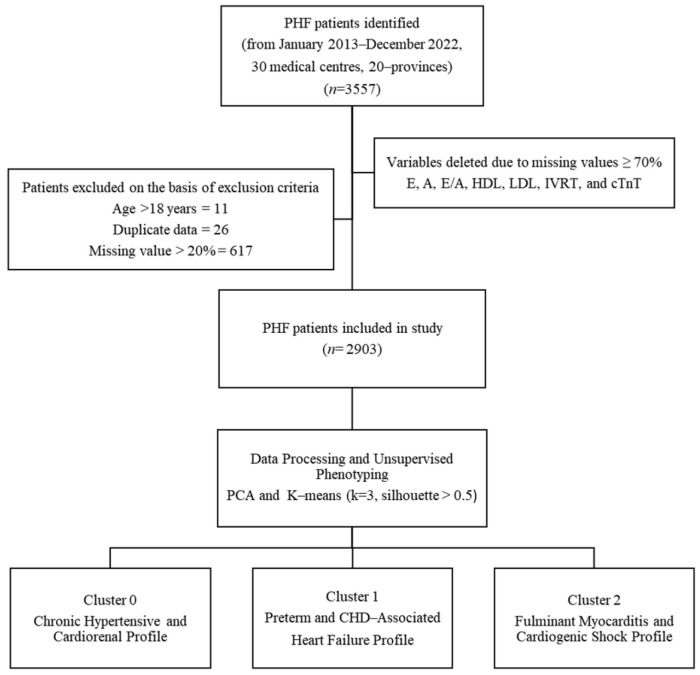
Patient Selection and Phenotyping Workflow. Flowchart of PHF patient selection from 30 Chinese medical centers (2013–2022). From an initial 3557 patients, exclusions included age > 18 years (*n* = 11), duplicates (*n* = 26), and records with >20% missing data (*n* = 617). The final cohort (*n* = 2903) underwent unsupervised phenotyping via principal component analysis (PCA) and k-means clustering (k = 3, silhouette score > 0.5), yielding three distinct phenotypes.

Our study cohort comprised pediatric inpatients meeting Chinese recommendations criteria for pediatric heart failure. It is important to note that these recommendations are harmonized with international guidelines (e.g., ISHLT, Modified Ross Classification) and are based on a combination of established clinical signs, symptoms, and objective echocardiographic and biomarker criteria to ensure a consistent and clinically relevant case definition. Exclusion criteria were systematically applied to mitigate bias: (1) incomplete medical records (≥20% missing data), (2) duplicate entries, (3) age > 18 years, and (4) mismatched admission-discharge timelines. After exclusions, the final analytical cohort included 2903 patients, with detailed attrition metrics documented in a flowchart (Figure 1).

### 2.4. Data Abstraction and Quality Assurance

We captured 99 clinically relevant variables spanning demographics and history (e.g., age, sex, gestational age, birth weight, history of CHD); vital signs and anthropometrics (e.g., weight, height, blood pressure); echocardiographic parameters (e.g., LVEF, chamber dimensions); laboratory biomarkers (e.g., BNP, creatinine); and pharmacotherapy (e.g., use of ACE inhibitors, diuretics) from our standardized electronic database (Table S2). Clinical and demographic data were extracted from hospital information systems (HIS) across all participating centers, with variables captured at the time of admission. In-hospital mortality, the primary outcome, was directly sourced from HIS records at the time of death; discharge status was similarly documented for survivors. To ensure accuracy, a standardized Microsoft Access database (Microsoft, Redmond, WA, USA) was employed, with trained personnel performing double-blinded data verification and entry.

### 2.5. Data Preprocessing and Feature Engineering

Clinically irrelevant or potentially confounding variables—including length of stay (LOS), financial metrics, and post-discharge parameters (e.g., LVEF at discharge)—were excluded a priori to prevent analytical bias. Variable selection was guided by clinical relevance to pediatric heart failure pathophysiology, spanning domains of demographics, clinical presentation, echocardiography, laboratory biomarkers, and comorbidities. This selection was based on expert consensus and alignment with established clinical guidelines and literature on pediatric heart failure. We prioritized variables that are routinely available in clinical practice and have demonstrated prognostic value in previous studies. Missing data were addressed through a tiered approach: first, variables with ≥70% missingness (E, A, EA, HDL, LDL, IVRT, cTnT) were removed entirely; second, patients with >20% missing data (*n* = 617) were excluded from the cohort; finally, any remaining missing values (<20% per record) in the final analytical cohort (*n* = 2903) were conservatively imputed using the median to preserve central tendency without introducing distributional skew. Imputation sensitivity analysis confirmed minimal deviation in core variables (Table S3), supporting the robustness of our imputation strategy. Continuous variables were standardized using z-score normalization (Scikit-learn’s StandardScaler) to eliminate scale-dependent artifacts in downstream machine learning.

### 2.6. Unsupervised Phenotyping via Dimensionality Reduction and Clustering

**Figure 2:**
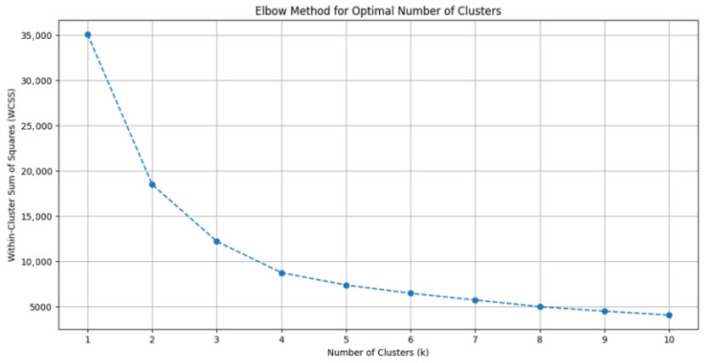
Determination of Optimal Cluster Number Using the Elbow Method. Plot of within-cluster sum of squares (WCSS) against the number of clusters for k-means clustering. The inflection point at k = 3 indicates optimal cluster selection, where additional clusters do not substantially improve variance explanation. This validated the three-phenotype model used in subsequent analyses.

Principal Component Analysis (PCA) was applied to reduce feature space dimensionality and mitigate multicollinearity risks while preserving clinically meaningful data structure. Optimal cluster count (k = 3) was determined via the Elbow Method, where the inflection point in the Within-Cluster Sum of Squares (WCSS) curve indicated maximal inter-cluster discrimination (Figure 2). K-means clustering was subsequently executed with 1000 random initializations to ensure reproducibility.

**Figure 3:**
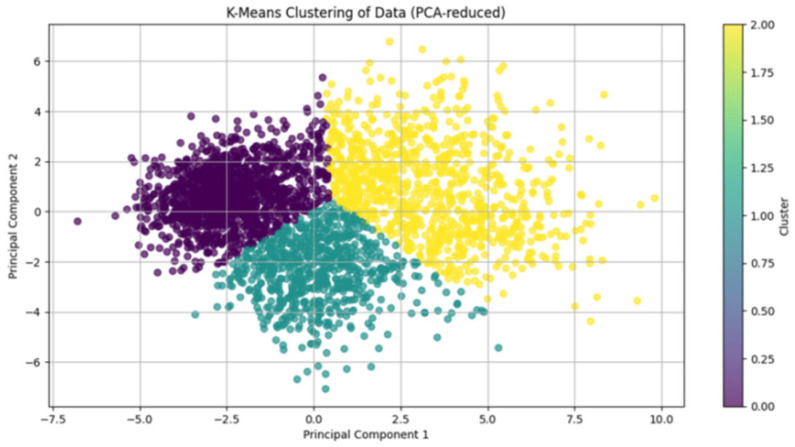
Visualization of K-Means Clustering in PCA-Reduced Feature Space. Two-dimensional projection of the PHF cohort using the first two principal components (PC1 and PC2) after PCA dimensionality reduction. Colors denote the three machine learning-derived phenotypes: Cluster 0 (purple), Cluster 1 (green), and Cluster 2 (yellow). The distinct spatial separation of clusters (silhouette score > 0.5) confirms robust phenotypic differentiation based on 99 clinical variables.

Cluster quality was validated using silhouette analysis, yielding an overall score of 0.58 (where >0.5 indicates reasonable structure separation; Figure 3). Phenotypic labels were derived by evaluating cluster-specific means of key variables against established heart failure subtyping frameworks for descriptive purposes.

### 2.7. Outcome Association Analysis

Cluster-outcome relationships were assessed through two primary endpoints: (1) in-hospital mortality (dichotomous) and (2) length of stay (LOS) categorized into clinically relevant intervals (<3, 3–7, 7–14, 15–30, >30 days). Both endpoints were analyzed using Pearson’s χ^2^ tests to evaluate phenotype-specific differences in frequency distributions. LOS comparisons employed χ^2^ tests of independence across the five predefined hospitalization duration strata. All analyses were conducted in Python 3.12 using Scikit-learn (v1.4.0) for machine learning, SciPy (v1.11.0) for hypothesis testing, and Matplotlib (v3.8.0) for visualization. Computational reproducibility was ensured through version-controlled Jupyter notebooks (v7.2.1) with randomized seed fixation (numpy.random.seed = 42).

## 3. Results

### 3.1. Demographic and Clinical Features Across Phenotypes

**Figure 4:**
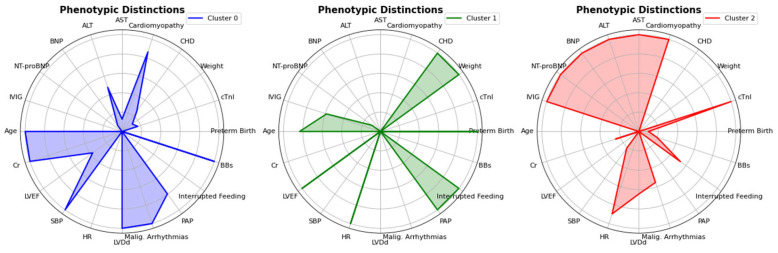
Radar Chart showing distinct phenotypic features. The radar charts depict the phenotypic distinctions between the three clusters identified in the analysis. Each chart compares the means of clinical variables, including age, BNP, LVEF, PAP, NT-proBNP, and others, for Cluster 0 (blue), Cluster 1 (green), and Cluster 2 (red). The differences in these variables highlight the unique phenotypic profiles associated with each cluster, providing insights into the clinical characteristics of the patient groups.

Unsupervised machine learning identified three phenotypically distinct clusters of PHF, subsequently classified based on their dominant clinical and pathophysiological features (Figure 4; Table S4).

Cluster 0 (“Chronic Hypertensive and Cardiorenal Profile”) predominantly affected older children (70.4%) and was characterized by hypertension (54.4%), renal dysfunction (creatinine 53.15 ± 39.67 μmol/L), and frequent ventricular tachycardia (53.8%). Cluster 1 (“Preterm and CHD-Associated HF”), representing preterm infants (71.4%) with congenital heart disease (72.2%), paradoxically showed preserved ejection fraction despite high mortality (5.08%). Cluster 2 (“Fulminant Myocarditis and Cardiogenic Shock Profile”) exhibited the most severe hemodynamic compromise (LVEF 35.26 ± 13.04%), multi-organ injury (ALT 121.17 ± 419.23 U/L), and distinctive treatment patterns (IVIG use 46.5%). It is important to note that these descriptive labels were assigned post hoc based on the predominant clinical and pathophysiological features that characterized each cluster. The labels are intended as a narrative aid to summarize the complex data patterns and do not imply direct causal mechanisms, which were not inferred by the unsupervised algorithm. These clusters with unique demographic, clinical, and etiological profiles are detailed in Table 1, highlighting both the heterogeneity of pediatric heart failure and the potential for phenotype-specific management strategies.

### 3.2. Clinical and Hemodynamic Parameters Across Phenotypes

Beyond clinical categorization, analysis of continuous variables revealed significant differences in anthropometric, hemodynamic, and cardiac function parameters across the three phenotypes (Table 2). Cluster 0 patients were significantly older and larger, with higher median weight (30.00 [21.00–41.42] kg) and height (140.00 [120.00–166.00] cm), along with elevated blood pressure (SBP 103.00 [94.00–112.00] mmHg, DBP 65.00 [58.00–73.00] mmHg) but lower heart rates. In contrast, Cluster 1 demonstrated marked growth restriction, yet preserved cardiac function (LVEF 67.00 [60.00–73.00]%, LVFS 36.00 [31.00–40.00]%) and the highest pulmonary artery pressures (49.00 [34.00–70.00] mmHg). Cluster 2 showed intermediate anthropometrics (weight 7.95 [6.22–11.50] kg, height 70.00 [62.00–84.00] cm) but the most severely impaired cardiac function (LVEF 33.00 [26.00–43.00]%, LVFS 17.00 [12.00–21.00]%) with compensatory tachycardia (140.00 [125.00–175.00] bpm). Chamber dimensions followed similar patterns, with Cluster 0 showing the largest cardiac structures (LA 28.00 [22.00–37.00] mm, LVDd 47.00 [38.00–57.00] mm) and Cluster 2 demonstrating disproportionate dysfunction relative to size.

### 3.3. Biomarkers Profile Across Phenotype

These structural and functional differences were further reflected in distinct biomarker profiles (Table 3). Cluster 2 demonstrated the most severe cardiac stress markers, with significantly elevated BNP and NT-proBNP levels (*p* < 0.001), suggesting profound ventricular strain. This cluster also showed evidence of multi-organ dysfunction, including higher hepatic enzymes (ALT, AST), and elevated cardiac injury markers including CK-MB, cTnI. Cluster 0 was characterized by renal dysfunction, i.e., creatinine, BUN, higher uric acid levels, and elevated hemoglobin. Cluster 1 showed relatively preserved biomarker profiles, with the lowest BNP levels and normal-range hepatic enzymes, though it had significantly higher platelet counts (326.00 [240.00–420.00] × 10^9^/L) compared to Cluster 0 (256.00 [197.00–321.25] × 10^9^/L). Oxygenation parameters differed significantly, with Cluster 2 paradoxically showing higher PO_2_ despite worse clinical severity. The marked biomarker differences between clusters provided biological rationale for their distinct treatment responses.

### 3.4. Treatment Patterns Across Phenotypes

These pathophysiological distinctions directly informed clinical management approaches (Table 4). Cluster 0 showed the highest utilization of beta-blockers (54.5%) and angiotensin-converting enzyme inhibitors (34.4%), consistent with its profile of chronic heart failure in older children. Cluster 1 demonstrated the most frequent use of diuretics (40.6% of total use) and antibiotics (54.3%), aligning with its predominance of congenital heart disease and associated fluid management needs. Cluster 2 stood out for its high utilization of intravenous immunoglobulin (46.5% of total IVIG use) and hormones (36.7%), reflecting the inflammatory and acute decompensation characteristics of this phenotype. Across all clusters, diuretics were the most commonly prescribed medication (overall 86.2%), highlighting fluid management as a universal priority in PHF, while inotropic agents showed relatively even distribution among phenotypes (27–31.4% of total use per cluster). These distinct treatment patterns underscore how clinical management naturally adapts to the different pathophysiological characteristics of each heart failure phenotype in clinical practice. However, it is important to note that these treatments represent in-hospital management decisions that may reflect both the underlying biology of each phenotype and institutional practice patterns or presenting acuity, rather than pre-existing biological characteristics.

### 3.5. Outcomes Across Phenotypes

The clinical relevance of these phenotypes was most evident in their distinct in-hospital outcomes (Table 5). Cluster 1, representing the largest subgroup (43.4%, *n* = 1261), demonstrated the highest in-hospital mortality rate (5.1%, 95% CI: 4.0–6.5%) and accounted for the majority of deaths (55.7%, *n* = 64). This group also showed a higher proportion of prolonged hospitalizations, with 10.6% of patients hospitalized for >30 days and 29.7% for 15–30 days, reflecting the complex clinical course of preterm and congenital heart disease patients. Cluster 2, comprising 26.5% (*n* = 769) of the cohort, exhibited an intermediate mortality rate (3.8%, 95% CI: 2.6–5.4%) but the highest acuity, with the shortest hospital stays (4.8% for <3 days) alongside a significant proportion of extended hospitalizations (40.7% for 7–14 days and 32.2% for 15–30 days), suggesting either rapid deterioration or prolonged recovery. Cluster 0, representing 30.1% (*n* = 873) of patients, had the lowest mortality (2.5%, 95% CI: 1.7–3.7%) and predominantly intermediate-length stays (23.0% for 3–7 days and 35.8% for 7–14 days), consistent with its characterization as a more stable, chronic condition. These distinct in-hospital mortality and hospitalization patterns strongly support the clinical utility of phenotyping for prognosis and resource allocation. All between-group differences were statistically significant (*p* < 0.001).

## 4. Discussion

PHF represents a complex syndrome where conventional classification systems have failed to capture the intricate interplay between developmental biology and disease pathophysiology. Our machine learning analysis of China’s largest multicenter PHF cohort has identified three clinically distinct phenotypes that redefine current diagnostic and therapeutic paradigms.

Our machine learning-derived phenotypes challenge the fundamental structure of current pediatric heart failure classifications, such as the modified ROSS score or NYHA-based systems, which primarily rely on symptomatic status and a single metric of systolic function (LVEF). While these traditional systems are clinically useful for gross stratification, they are inherently reductionist. For instance, Cluster 1 (“Preterm and CHD-Associated HF”) exemplifies this limitation: these patients exhibited the highest mortality (5.1%) despite having preserved LVEF (67%), a group that would typically be categorized as lower risk in an LVEF-centric paradigm. Our approach, by integrating 99 variables spanning demographics, biomarkers, imaging, and comorbidities, captures the pathophysiological complexity that symptom scores or ejection fraction alone cannot. This is particularly critical for identifying high-risk subgroups like Cluster 1, where the risk stems from myocardial immaturity, pulmonary hypertension, and comorbidities rather than isolated pump failure.

To illustrate the potential clinical applicability of our phenotyping framework, consider how early recognition of these distinct profiles could guide differential management pathways. For patients identified with the Fulminant Myocarditis phenotype (Cluster 2), the clinical pathway would prioritize immediate immunomodulation with intravenous immunoglobulin, early consideration of mechanical circulatory support, and intensive hemodynamic monitoring [24,25]. In contrast, patients with the Preterm and CHD-Associated phenotype (Cluster 1) would benefit from a fundamentally different approach focused on meticulous fluid balance management, pulmonary vasodilator therapy, nutritional optimization, and infection prophylaxis, addressing their unique pathophysiology of myocardial immaturity and chronic volume overload [6]. Meanwhile, the Chronic Hypertensive and Cardiorenal phenotype (Cluster 0) would warrant emphasis on arrhythmia surveillance, renal-protective antihypertensive regimens, and long-term cardiovascular risk modification. These examples demonstrate how phenotype-specific recognition at presentation could streamline clinical decision-making, optimize resource allocation, and ultimately enable more personalized care delivery in pediatric heart failure.

From a practical standpoint, while the implementation of an ML model in real-time clinical decision-making requires further validation and integration into health information systems, the immediate utility of our framework lies in its ability to reframe PHF as distinct biological entities. This phenotyping could inform future resource allocation planning and provides a rationale for the design of targeted clinical trials. It is critical to emphasize that our analysis is observational. The associations between phenotypes and specific treatment patterns reflect real-world clinical practice rather than evidence of treatment efficacy. Therefore, the phenotypic profiles and their correlated therapies should be viewed as hypothesis-generating, forming a foundation for future prospective studies and interventional trials to determine if phenotype-guided management truly improves outcomes.

Cluster 0 represents a clinically distinct form of pediatric heart failure that challenges conventional management paradigms. Our analysis reveals this phenotype predominantly affects older children and is characterized by a triad of chronic hypertension, significant arrhythmic burden, and renal dysfunction. This aligns with previous studies, which reported that hypertensive heart failure in older children is associated with malignant arrhythmia and secondary renal impairment [6,26,27,28]. While demonstrating the lowest mortality among our clusters, this phenotype carries substantial long-term morbidity risks. The high prevalence of malignant arrhythmia and renal impairment suggests these patients may be particularly vulnerable to sudden cardiac events and progressive end-organ damage, despite preserved systolic function. This finding aligns with hypertensive heart disease patterns, where electrical instability often precedes pump failure [29].

Similarly, Cluster 1 (“Preterm and CHD-Associated HF with High-Risk Compensated Physiology”) encapsulates the complex interplay between congenital heart disease (CHD) and developmental immaturity that defines the most vulnerable PHF population. The high mortality rate in this cluster is directly attributable to this synergy, with CHD acting as the primary driver of hemodynamic insult and prematurity compounding the risk by imposing a substrate of myocardial and pulmonary immaturity, consistent with prior work [1,30,31,32,33]. Our findings not only confirm CHD as the dominant etiology but also crucially reveal how prematurity modifies both disease presentation and outcomes. Previous studies relied on NYHA classification or LVEF to grade severity [23,34,35]. The paradoxical combination of preserved systolic function with high mortality in Cluster 1 suggests that LVEF alone may be insufficient for comprehensive risk stratification in this specific population of preterm infants with CHD. However, given the observational nature of our study and the clustering of other high-risk features (prematurity, CHD complexity) in this phenotype, we cannot determine whether preserved EF is intrinsically misleading or simply collinear with these other risk factors. This finding underscores the need for pediatric-specific metrics that incorporate right ventricular function and pulmonary vascular interactions but requires validation in studies designed to test the independent prognostic value of EF in this population. The prolonged hospitalizations reflect the unique pathophysiology of this phenotype, where immature myocardium struggles to adapt to abnormal loading conditions. These results extend beyond previous CHD studies by demonstrating that prematurity status and CHD complexity create distinct clinical trajectories that conventional classification systems fail to capture.

Cluster 2 (“Fulminant Myocarditis and Cardiogenic Shock Profile”) represents a distinct and clinically critical phenotype that challenges conventional understanding of pediatric cardiogenic shock. While previous studies have described acute heart failure in children as a uniform entity with a poor prognosis, our findings reveal important nuances in disease trajectory and therapeutic response. The profound myocardial dysfunction observed in this cluster aligns with prior reports of fulminant myocarditis in pediatric populations [36,37], yet the intermediate mortality rate contrasts with the uniformly poor outcomes typically reported in adult cardiogenic shock studies [38,39]. This discrepancy may reflect fundamental differences in myocardial resilience between children and adults, as suggested by recent work on pediatric myocardial recovery mechanisms [40].

The strong inflammatory signature of this phenotype, suggested by its distinctive treatment pattern favoring immunomodulatory therapies, is consistent with the role of cytokine storms in heart failure. Several recent studies have highlighted the potential benefits of early immunomodulation in pediatric myocarditis, though none have identified this as a distinct phenotypic subgroup prior to our work [41,42,43,44]. The pediatric-specific characteristics of this phenotype may explain why some children demonstrate remarkable recovery despite initially severe presentations, a phenomenon that has been anecdotally reported but never systematically characterized. This cluster’s identification provides a framework for re-evaluating therapeutic approaches in pediatric acute heart failure, particularly regarding the timing of advanced support and immunomodulation. When interpreting the treatment patterns associated with each phenotype, it is crucial to recognize that these represent real-world clinical responses to each cluster’s presentation rather than defining features of the phenotypes themselves. The observed patterns likely reflect a combination of the underlying pathophysiology and institutional treatment preferences, and we cannot definitively separate these influences in our retrospective design.

The phenotypic distinctions we identified likely reflect fundamental differences in disease mechanisms across developmental stages. For Cluster 0, the hypertensive-arrhythmic-renal triad suggests unique pediatric manifestations of neurohormonal activation, potentially involving age-dependent patterns of angiotensin II receptor expression [45] and myocardial fibrosis deposition [46]. The preserved systolic function despite electrical instability may relate to developmental differences in calcium-handling proteins [47]. Cluster 1 exemplifies the collision of myocardial immaturity with congenital hemodynamic insults. Preterm infants exhibit underdeveloped sarcoplasmic reticulum and t-tubule systems [48], rendering them vulnerable to volume overload from CHD. This aligns with emerging evidence that myocardial stiffness correlates with abnormal titin isoform ratios [49], potentially explaining their preserved EF despite clinical decompensation. The pulmonary vascular dysfunction we observed may stem from disrupted alveolar-capillary development [50], creating a “double-hit” of right ventricular pressure overload and impaired ventricular interdependence [51]. These insights provide a rationale for investigating therapies that address both myocardial immaturity and pulmonary vascular disease in this high-risk population. Cluster 2 demonstrates the complex interplay between inflammatory cascades and developmental cardiac resilience. Myocardium exhibits enhanced capacity for autophagy-mediated recovery after inflammatory injury [52], which may explain the bimodal outcomes we observed. The IVIG response patterns warrant investigation of age-specific immunomodulatory strategies.

Our framework could inform the development of phenotype-specific management strategies grounded in developmental pathophysiology. For example, in Cluster 0, the high arrhythmic burden suggests that future studies could evaluate the utility of early ambulatory arrhythmia monitoring combined with renal-sparing antihypertensives. The efficacy of beta-blockers in this group supports recent findings of pediatric-specific β-adrenergic receptor maturation patterns [53], suggesting potential for pharmacogenomics targeting. Cluster 1 patients may benefit from pulmonary vasodilators timed to myocardial maturation windows, as suggested by an experimental study of treprostinil in preterm ventricular mechanics [54]. The prolonged hospitalizations underscore the need for nutritional strategies addressing preterm metabolic demands during CHD recovery. For Cluster 2, the inflammatory signature and treatment patterns suggest that tiered immunomodulation protocols, including early IVIG, should be evaluated in prospective trials. The bimodal recovery pattern suggests mechanical support algorithms (e.g., ECMO [Extracorporeal Membrane Oxygenation]) should incorporate inflammatory markers rather than relying solely on hemodynamic parameters.

The prolonged hospitalizations in Cluster 1 carry significant resource utilization implications, highlighting an area where phenotype-specific care pathways might be explored for efficiency. Conversely, Cluster 2’s bimodal outcomes (shortest/longest LOS in Table 5) align with its inflammatory pathophysiology, suggesting that rapid responders might be identified for early IVIG, while prolonged recoverers may be candidates for sustained immunomodulation in future studies. For research, these phenotypes could enable smarter trial design by reducing clinical heterogeneity. Cluster 2 is particularly suited for trials of immunomodulatory regimens, while Cluster 1 represents a candidate population for trials of pulmonary vasodilators in CHD patients. The molecular characterization of each phenotype may reveal novel therapeutic targets and biomarkers.

### 4.1. Limitations

Our study has several limitations. First, our study cohort was derived largely from tertiary care centers within China’s healthcare system. While this provided a large sample of complex cases, it may limit the generalizability of our findings to non-tertiary settings or other populations with different healthcare access and practices. Furthermore, while the Chinese diagnostic criteria used are aligned with international standards, the possibility that minor differences in practice could influence case ascertainment compared to other regions cannot be entirely ruled out. Nevertheless, although our multicenter design enhances generalizability, prospective validation in diverse populations is needed. Third, the exclusion of patients with >20% missing data, though necessary for model stability, may have led to an under-representation of the most critically ill and complex phenotypes, potentially making the derived clusters appear more distinct than they would in a real-world, unselected cohort. Fourth, the stability of the identified phenotypes was not formally tested against alternative preprocessing strategies. While this is a methodological consideration, the risk of the clusters being analytical artifacts is substantially mitigated by the use of standardized techniques, the minimal imputation required, and—most critically—the strong prognostic discrimination of the phenotypes, which is unlikely to emerge from a spurious cluster structure. Fifth, the prognostic value of our phenotypes is limited to in-hospital outcomes. Their association with long-term post-discharge events remains unknown and requires future investigation. The retrospective nature limited our ability to capture some potential confounders like detailed medication dosing or genetic factors.

### 4.2. Future Directions

Building upon these findings, several critical research directions emerge. Future studies should prioritize prospective validation of these phenotypes in diverse populations and healthcare settings to establish generalizability. Longitudinal follow-up is essential to assess phenotype stability and long-term outcomes beyond the in-hospital period. Incorporating advanced imaging modalities and omics profiling will help characterize the myocardial substrate and uncover underlying molecular mechanisms for each phenotype. Most importantly, intervention trials are needed to test phenotype-specific management protocols, such as early immunomodulation in fulminant myocarditis or pulmonary vasodilators in preterm and CHD-associated heart failure.

## 5. Conclusions

In conclusion, our machine learning analysis of PHF patients identified three distinct phenotypes—the chronic hypertensive and cardiorenal profile, the preterm and CHD-associated profile, and the fulminant myocarditis and cardiogenic shock profile. These data-driven phenotypes underscore the imperative for phenotype-specific management, from arrhythmia monitoring in older children to immunomodulation in inflammatory shock. This framework advances the understanding of PHF heterogeneity and provides a foundational step toward precision medicine. Future research should focus on the prospective and external validation of these phenotypes to assess their generalizability and utility in guiding clinical care.

## Figures and Tables

**Table 1:** Demographic, Clinical, Etiological and Diagnostics Features across PHF Phenotypes.

Variable	Overall *n* (%)	Cluster 0 *n* (%)	Cluster 1 *n* (%)	Cluster 2 *n* (%)	*p*-Value
Demographic features					
Sex Male	1501 (51.7)	459 (30.6%)	683 (45.5%)	359 (23.9)	0.004
Age Group					<0.001
Neonatal	212 (7.3)	1 (0.5)	191 (90.1)	20 (9.4)	
Infant and toddler	1618 (55.7)	32 (1.98)	987 (61)	599 (37.02)	
Child	766 (26.4)	539 (70.4)	83 (10.8)	144 (18.8)	
Teenager	307 (10.6)	301 (98)	0 (0)	6 (2)	
Birth Type					<0.001
Preterm birth	315 (28.9)	36 (11.4)	225 (71.4)	54 (17.1)	
Term birth	762 (69.8)	117 (15.4)	445 (58.4)	200 (26.2)	
Post-term birth	14 (1.3)	3 (21.4)	3 (21.4)	8 (57.2)	
BMI					<0.001
Underweight	571 (26.9)	210 (36.8)	220 (38.5)	141 (24.7)	
Normal	1383 (65)	415 (30)	635 (45.9)	333 (24.1)	
Overweight	173 (8.1)	31 (17.9)	93 (53.8)	49 (28.3)	
Clinical Features					
Blood Pressure					<0.001
Normal	1987 (78.1)	583 (29.3)	839 (42.2)	565 (28.5)	
Hypotension	152 (5.9)	47 (31)	63 (41.4)	42 (27.6)	
Hypertension	406 (16)	221 (54.4)	106 (26.1)	79 (19.5)	
Modified ROSS Classification					0.001
I, II	783 (37.5)	282 (36)	317 (40.5)	184 (23.5)	
III, IV	1308 (62.5)	449 (34.3)	458 (35)	401 (30.7)	
Respiratory symptoms	2185 (75.3)	505 (23.1)	1085 (49.7)	595 (27.2)	<0.001
Gastrointestinal symptoms	717 (24.7)	317 (44.2)	179 (25)	221 (30.8)	<0.001
Systemic Venous Congestion	2153 (74.2)	525 (24.4)	1000 (46.4)	628 (29.2)	<0.001
Interrupted feeding	816 (28.1)	1 (0.1)	533 (65.3)	282 (34.6)	<0.001
Pallor	867 (30)	206 (23.8)	355 (40.9)	306 (35.3)	<0.001
Restlessness	613 (21.1)	65 (10.6)	326 (53.2)	222 (36.2)	<0.001
HF type and etiology					
AHF	1801 (63.6)	476 (26.4)	801 (44.5)	524 (29.1)	<0.001
CHF	1029 (36.4)	371 (36.1)	426 (41.4)	232 (22.5)	
Congenital Heart Disease (CHD)	1062 (36.6)	150 (14.1)	767 (72.2)	145 (13.7)	<0.001
Simple CHD	331 (11.4)	43 (13)	244 (73.7)	44 (13.3)	<0.001
Complex CHD	731 (21.2)	107 (14.6)	523 (71.5)	101 (13.8)	<0.001
ASD	515 (17.7)	125 (24.3)	219 (42.5)	171 (33.2)	<0.001
VSD	427 (14.7)	109 (25.5)	182 (42.6)	136 (31.9)	0.011
PDA	297 (10.2)	73 (24.6)	130 (43.8)	94 (31.6)	0.036
Cardiomyopathy	978 (33.7)	429 (43.9)	61 (6.2)	488 (49.9)	<0.001
HCM	62 (2.1)	27 (43.5)	23 (37.1)	12 (19.4)	0.06
DCM	463 (16)	149 (32.2)	220 (47.5)	94 (20.3)	0.004
RCM	47 (1.61)	13 (27.7)	25 (53.2)	9 (19.1)	0.348
ARVC	57 (2)	20 (35.1)	27 (47.4)	10 (17.5)	0.294
Cardiac and Radiological findings					
Myocardial densification insufficiency	229 (7.9)	72 (31.4)	94 (41)	63 (27.5)	0.748
Endocardial elasto-fibrillar hyperplasia	155 (5.3)	58 (37.4)	64 (41.3)	33 (21.3)	0.091
Infection	850 (29.3)	201 (23.6)	442 (52)	207 (24.4)	<0.001
Cardiomegaly	1794 (71.6)	578 (32.2)	638 (35.6)	578 (32.2)	<0.001
Pulmonary Congestion	853 (36.6)	294 (34.5)	368 (43.1)	191 (22.4)	<0.001
Pulmonary Hypoperfusion	14 (0.6)	2 (14.3)	4 (28.6)	8 (57.1)	0.032
Prominent aortic node	9 (0.4)	5 (55.6)	3 (33.3)	1 (11.1)	0.219
Prominent pulmonary artery segment	73 (3.1)	23 (31.5)	30 (41.1)	20 (27.4)	0.895
Supraventricular tachycardia	281 (1)	116 (41.3)	89 (31.7)	76 (27)	<0.001
Ventricular tachycardia	169 (6.1)	91 (53.8)	35 (20.7)	43 (25.4)	<0.001
Malignant arrhythmias	167 (6.1)	73 (43.7)	37 (22.2)	57 (34.1)	<0.001

AHF: Acute heart failure, CHF: Chronic heart failure, ASD: Atrial septal defect, VSD: Ventricular septal defect, PDA: Patent Ductus Arteriosus, HCM: Hypertrophic cardiomyopathy, RCM: Restrictive cardiomyopathy, DCM: Dilated Cardiomyopathy, ARVC: Arrhythmogenic Right Ventricular Cardiomyopathy, Demographic, clinical, and etiological characteristics of pediatric heart failure patients, stratified by phenotypic clusters (Cluster 0, Cluster 1, Cluster 2). Values are represented as *n* (%) and *p*-values were derived from Pearson’s Chi-squared test of independence for comparisons of categorical variables across clusters.

**Table 2:** Clinical and Hemodynamic Parameters across PHF Phenotypes.

Variable	Overall Median (IQR)	Cluster 0 Median (IQR)	Cluster 1 Median (IQR)	Cluster 2 Median (IQR)	*p*-Value
Gestational Week	38.57 [37.00–39.86]	39.00 [37.97–40.00]	38.14 [36.29–39.57]	39.00 [37.71–40.00]	<0.001
Birth weight, kg	3.20 [2.90–3.50]	3.25 [3.00–3.56]	3.10 [2.70–3.49]	3.25 [3.00–3.53]	<0.001
Weight, kg	8.75 [5.50–20.23]	30.00 [21.00–41.42]	5.50 [4.00–8.00]	7.95 [6.22–11.50]	<0.001
Height, cm	73.00 [60.00–119.00]	140.00 [120.00–166.00]	60.00 [54.00–70.00]	70.00 [62.00–84.00]	<0.001
SBP, mmHg	91.00 [82.00–104.00]	103.00 [94.00–112.00]	85.00 [78.00–94.00]	89.00 [81.00–97.00]	<0.001
DBP, mmHg	56.00 [48.00–65.00]	65.00 [58.00–73.00]	50.00 [43.00–59.00]	53.00 [46.00–61.00]	<0.001
HR, bpm	135.00 [114.00–168.00]	106.00 [90.00–122.00]	145.00 [130.00–180.00]	140.00 [125.00–175.00]	<0.001
LA, mm	19.00 [16.00–25.00]	28.00 [22.00–37.00]	17.00 [12.75–19.00]	20.00 [17.00–24.00]	<0.001
RA, mm	23.00 [18.00–31.00]	35.00 [28.00–44.00]	19.00 [16.00–22.00]	22.00 [18.00–26.00]	<0.001
RV, mm	14.00 [11.00–20.00]	21.00 [17.00–29.00]	13.00 [10.00–18.00]	13.00 [10.00–17.00]	<0.001
LVDd, mm	34.00 [24.00–44.00]	47.00 [38.00–57.00]	24.00 [19.00–29.00]	39.00 [33.00–45.00]	<0.001
LVDs, mm	22.00 [16.00–34.00]	34.00 [24.00–47.00]	16.00 [12.00–19.00]	32.00 [26.00–38.00]	<0.001
AO, mm	13.00 [11.00–18.00]	19.00 [18.00–22.00]	11.00 [10.00–13.00]	12.00 [11.00–14.00]	<0.001
IVDd, mm	5.00 [4.00–7.00]	7.00 [6.00–8.00]	4.00 [4.00–5.00]	5.00 [4.00–6.00]	<0.001
LVPWD, mm	5.00 [4.00–6.00]	6.00 [6.00–8.00]	4.00 [3.00–5.00]	5.00 [4.00–6.00]	<0.001
LVEF, %	54.00 [35.00–68.00]	46.00 [32.00–60.00]	67.00 [60.00–73.00]	33.00 [26.00–43.00]	<0.001
LVFS, %	27.00 [18.00–36.00]	23.00 [17.00–31.00]	36.00 [31.00–40.00]	17.00 [12.00–21.00]	<0.001
PAP, mmHg	42.00 [29.00–60.25]	42.00 [30.00–61.00]	49.00 [34.00–70.00]	33.00 [22.00–45.50]	<0.001

SBP: Systolic Blood Pressure, DBP: Diastolic Blood Pressure, HR: Heart Rate, LA: Left Atrium, RA: Right Atrium, RV: Right Ventricle, LVDd: Left Ventricular End-Diastolic Diameter, LVDs: Left Ventricular End-Systolic Diameter, AO: Aortic Diameter, IVDd: Intraventricular Septum End-Diastolic Diameter, LVPWD: Left Ventricular Posterior Wall Diameter, LVEF: Left Ventricular Ejection Fraction, LVFS: Left Ventricular Fractional Shortening, PAP: Pulmonary Artery Pressure.This table presents clinical hemodynamic parameters in pediatric heart failure patients. The values are reported as median (IQR), with significant differences between phenotypic clusters noted by *p*-values. *p*-values were derived from Kruskal–Wallis H tests for comparisons of continuous variables across clusters.

**Table 3:** Distinct Biomarker Profiles across PHF Phenotypes.

Variable	Overall Median (IQR)	Cluster 0 Median (IQR)	Cluster 1 Median (IQR)	Cluster 2 Median (IQR)	*p*-Value
BNP, pg/mL	935.00 [182.00–3509.25]	820.00 [211.50–2251.00]	466.50 [110.00–1938.25]	3234.00 [1039.00–5000.00]	<0.001
NT-proBNP, pg/mL	5690.50 [1753.00–18,004.75]	4293.00 [1179.00–10,660.00]	3823.50 [1026.75–13,389.75]	14,448.00 [5327.50–30,000.00]	<0.001
CK-MB, µg/L	7.30 [2.80–23.16]	5.70 [2.12–20.00]	6.85 [3.20–23.26]	10.20 [3.58–27.00]	<0.001
cTnI, µg/L	0.06 [0.01–0.28]	0.04 [0.01–0.19]	0.06 [0.02–0.23]	0.14 [0.03–0.48]	<0.001
ALT, U/L	26.00 [17.60–46.27]	23.00 [14.55–41.00]	27.00 [18.00–43.35]	28.00 [18.00–56.28]	<0.001
AST, U/L	43.60 [31.48–65.78]	35.00 [25.00–55.00]	44.90 [33.60–64.00]	49.00 [37.00–84.00]	<0.001
ALB, g/L	39.40 [34.90–43.10]	39.80 [35.10–43.20]	39.10 [34.20–42.90]	39.40 [35.90–43.10]	0.02
ALP, U/L	189.00 [134.00–264.00]	179.65 [123.23–228.25]	203.00 [142.00–289.00]	188.30 [134.38–259.25]	<0.001
Cr, µmol/L	30.30 [23.00–45.00]	45.80 [36.00–59.45]	25.00 [20.00–33.00]	28.20 [22.00–38.00]	<0.001
BUN, mg/dL	4.50 [3.07–6.22]	5.30 [4.15–7.04]	3.60 [2.50–5.28]	4.75 [3.22–6.59]	<0.001
UA, µmol/L	311.00 [218.00–430.00]	393.15 [296.73–519.00]	246.60 [180.00–332.30]	343.55 [253.00–473.35]	<0.001
Sodium, mmol/L	138.00 [135.00–140.00]	138.00 [136.00–140.00]	138.00 [135.00–140.00]	137.00 [134.00–139.00]	<0.001
WBC, ×10^9^/L	9.09 [6.88–11.91]	8.30 [6.50–11.00]	9.62 [7.27–12.50]	9.18 [6.90–12.20]	<0.001
RBC, ×10^12^/L	4.22 [3.70–4.73]	4.60 [4.17–5.01]	4.04 [3.50–4.63]	4.08 [3.63–4.46]	<0.001
PLT, ×10^9^/L	303.00 [221.00–389.00]	256.00 [197.00–321.25]	326.00 [240.00–420.00]	334.00 [244.00–412.00]	<0.001
Hb, g/dL	116.00 [102.00–130.00]	127.00 [117.00–138.75]	112.00 [99.00–126.00]	108.00 [96.00–119.00]	<0.001
MCV, fL	85.00 [80.00–90.00]	85.00 [81.00–88.00]	86.00 [80.00–94.00]	83.00 [78.00–88.00]	<0.001
MCH, pg	28.00 [26.00–30.00]	28.00 [27.00–30.00]	28.00 [26.00–31.00]	27.00 [25.00–29.00]	<0.001
MCHC, g/dL	328.00 [318.00–337.00]	330.00 [321.00–338.00]	327.00 [317.00–337.00]	325.00 [314.00–336.00]	<0.001
PT, s	14.00 [12.00–16.00]	14.00 [12.00–16.00]	13.00 [12.00–15.00]	14.00 [13.00–17.00]	<0.001
APTT, s	34.00 [29.00–40.00]	32.00 [28.00–36.00]	35.00 [29.00–42.00]	33.00 [28.00–39.00]	<0.001
PO_2_, mmHg	78.00 [46.20–110.70]	75.10 [43.06–108.00]	72.00 [48.00–100.00]	91.10 [49.05–135.00]	<0.001
PCO_2_, mmHg	37.00 [31.10–44.12]	35.00 [30.10–40.10]	40.50 [33.30–49.40]	35.00 [29.32–40.80]	<0.001

BNP: B-type Natriuretic Peptide, NT-proBNP: N-terminal pro-B-type Natriuretic Peptide, CK-MB: Creatine Kinase-Myocardial Band, cTnI—Cardiac Troponin I, ALT: Alanine Aminotransferase, AST: Aspartate Aminotransferase, ALB: Albumin, ALP: Alkaline Phosphatase, Cr: Creatinine, BUN: Blood Urea Nitrogen, UA: Uric Acid, WBC: White Blood Cells, RBC: Red Blood Cells, PLT: Platelets, Hb: Hemoglobin, MCV: Mean Corpuscular Volume, MCH: Mean Corpuscular Hemoglobin, MCHC: Mean Corpuscular Hemoglobin Concentration, PT: Prothrombin Time, APTT: Activated Partial Thromboplastin Time, PO_2_: Partial Pressure of Oxygen, PCO_2_: Partial Pressure of Carbon Dioxide. The values are reported as median (IQR), with significant differences between phenotypic clusters noted by *p*-values. *p*-values were derived from Kruskal–Wallis H tests for comparisons of continuous variables across clusters.

**Table 4:** Phenotype-Specific Pharmacological Treatment Patterns in PHF.

Variable	Overall *n* (%)	Cluster 0 *n* (%)	Cluster 1 *n* (%)	Cluster 2 *n* (%)	*p*-Value
ACEIs	1300 (44.8)	447 (34.4)	314 (24.2)	539 (41.4)	<0.001
BBs	486 (16.7)	265 (54.5)	94 (19.3)	127 (26.2)	<0.001
Diuretics	2504 (86.2)	758 (30.3)	1016 (40.6)	730 (29.2)	<0.001
IA	2371 (81.7)	640 (27)	987 (41.6)	744 (31.4)	<0.001
Antibiotics	2008 (69.2)	405 (20.2)	1091 (54.3)	512 (25.5)	<0.001
Hormones	1355 (46.7)	284 (21)	573 (42.3)	498 (36.7)	<0.001
IVIG	862 (29.7)	157 (18.2)	304 (35.3)	401 (46.5)	<0.001

ACEIs: Angiotensin-Converting Enzyme Inhibitors, IVIG: Intravenous Immunoglobulin, BBs: Beta-Blockers, IA: Inotropic Agents. Values are represented as *n* (%) and *p*-values were derived from Pearson’s Chi-squared test of independence for comparisons of categorical variables across clusters.

**Table 5:** Mortality and Hospitalization Outcomes across Phenotypes.

Variable	Cluster 0	Cluster 1	Cluster 2	*p*-Value
Total Patients *n* (%)	873 (30.1)	1261 (43.4)	769 (26.5)	<0.001
Number of Deaths	22 (19.1)	64 (55.7)	29 (25.2)	
Death Rate (%) (95% CI)	2.5 (1.7–3.7)	5.1 (4.0–6.5)	3.8 (2.6–5.4)	
Length of Stay count (Patients) *n* (%)	865 (30.1)	1252 (43.6)	757 (26.3)	<0.001
<3 days	47 (5.4)	71 (5.7)	36 (4.8)	
3–7 days	199 (23)	213 (17)	91 (12)	
7–14 days	310 (35.8)	463 (37)	308 (40.7)	
15–30 days	241 (27.9)	372 (29.7)	244 (32.2)	
>30 days	68 (7.9)	133 (10.6)	78 (10.3)	

Mortality and length of stay (LOS) outcomes across phenotypic clusters. LOS is categorized into intervals (<3 days, 3–7 days, 7–14 days, 15–30 days, >30 days), Values are represented as *n* (%), 95% CI was measured using Wilson score interval method, and the *p*-values were derived from Pearson’s Chi-squared tests of independence. The total number of patients analyzed for Length of Stay (LOS) is lower than the total cohort because patients with missing LOS data were excluded. Specifically, 8 (Cluster 0), 9 (Cluster 1), and 12 (Cluster 2) patients were excluded from the LOS analysis.

## Data Availability

The original contributions presented in this study are included in the article/Supplementary Materials. Further inquiries can be directed to the corresponding authors.

## Supplementary Materials

The following supporting information can be downloaded at: https://www.mdpi.com/article/10.3390/diagnostics15222893/s1, Table S1. List of Participating Institutions and Regions. Table S2. Variables included in the Study. Table S3. Core Variables Pre-Post Median imputation Sensitivity Analysis. Table S4. Top 20 Discriminating Variables Across Pediatric HF Phenotypes. Table S5. List of researchers (Arranged from high to low according to their contribution).
