# Supplementary material for: Machine Learning Reveals Novel Pediatric Heart Failure Phenotypes with Distinct Mortality and Hospitalization Outcomes
Source: Diagnostics (Basel). 2025 Nov 14;15(22):2893. doi: 10.3390/diagnostics15222893 (PMC12650898; doi:10.3390/diagnostics15222893)
Supplement: Supplementary file 1 [file diagnostics-15-02893-s001.zip › diagnostics-3941312-supplementary.pdf]

Table S1. List of Participating Institutions and Regions

Province or Municipality	Center
Beijing City	Fuwai hospital，Chinese Academy of Medical Sciences, Peking Union Medical College
	Beijing An Zhen Hospital of the Capital University of Medical Sciences
Tianjin City	Tianjin Children's Hospital
Hebei Province	Hebei Children's Hospital
	The Second Hospital of Hebei Medical University
Inner Mongolia Autonomous Region	Inner Mongolia People's Hospital
Liaoning Province	Shengjing Hospital of China Medical University
Jilin Province	The First Bethune Hospital of Jilin University
Shanghai City	Children's Hospital of Fudan University
	Shanghai Children’s Medical Center, School of Medicine, Shanghai Jiao Tong University
	Xinhua Hospital Affiliated to Shanghai Jiaotong University School of Medicine
Jiangsu Province	Children's Hospital of Nanjing Medical University
Zhejiang Province	Children's Hospital, Zhejiang University School of Medicine
Anhui Province	Anhui Provincial Hospital
Jiangxi Province	Jiangxi Children's Hospital
Shandong Province	Qingdao Women and Children's Hospital
	Children's Hospital Affiliated to Shandong University, Jinan Children's Hospital
	Qilu Hospital of Shandong University
Henan Province	Henan Children's Hospital, Children's Hospital Affiliated to Zhengzhou University
	The First Affiliated Hospital of Zhengzhou University
Hunan Province	Hunan Children's Hospital
Guangdong Province	Guangdong Provincial People's Hospital, Guangdong Academy of Medical Sciences
	Guangzhou Women and Children's Medical Center，Guangzhou Medical University
Chongqing City	Children’s Hospital of Chongqing Medical University
Sichuan Province	Sichuan Academy of Medical Sciences & Sichuan Provincial People's Hospital
	Sichuan Provincial Maternity and Child Health Care Hospital
	The Affiliated Hospital of Southwest Medical University
Tibet Autonomous Region	Tibet Autonomous Region People's Hospital
Shaanxi Province	Xi'an Children's Hospital
Xinjiang Uygur Autonomous Region	The First Affiliated Hospital of Shihezi

The list is presented without any specific order or ranking.

Table S2. Variables included in the Study

Category	Variable Count	List of Variables
**Demographic & History**	13	Demographic: Sex, Ethnic, Age, Age in months, Age in years, Age Group, GW (Gestational Weeks), Pre-term, BW (Birth Weight), Weight, Height, BMI<br>Medical History: Infection History
**Signs & Symptoms**	12	Vital Signs: SBP, DBP, Blood Pressure levels, HR<br>Clinical Signs: Malignant arrythmias, Reg (Regurgitation)<br>Symptom Complexes: SymA (Respiratory), SymB (Gastrointestinal), SymC (Systemic Venous Congestion), SymD (Interrupted Feeding), SymE (Pallor), SymF (Restlessness)
**Clinical Classifications**	15	Modified ROSS classification, HF type (AHF/CHF), CHD (Congenital Heart Disease - overall), CM (Cardiomyopathy - overall)<br>Sub-types:<br>- CHD Sub-types: SCHD (Simple CHD), CCHD (Complex CHD), CHD1 (ASD), CHD2 (VSD), CHD3 (PDA)<br>- CM Sub-types: CM1 (HCM), CM2 (DCM), CM3 (RCM), CM4 (Myocardial densification), CM5 (Endocardial elastofibrillar hyperplasia), CM6 (Arrhythmogenic RV cardiomyopathy)
**Diagnostics (Lab Tests)**	29	BNP, NPBNP, CKMB, cTnI, ALT, AST, ALB, ALP, Cr, BUN, UA, K, Na, Ca, P, WBC, RBC, PLT, Hb, MCV, MCH, MCHC, PT, APTT, PO2, PCO2, TG, TC, FPG
**Auxiliary Examinations (Imaging/Procedures)**	18	Imaging Findings (X-ray): Xry1 (Cardiomegaly), Xry2 (Pulmonary Congestion), Xry3 (Pulmonary Hypoperfusion), Xry4 (Prominent aortic node), Xry5 (Prominent pulmonary artery segment)<br>Electrocardiogram: SVT, VT<br>Echocardiography: LA, RA, RV, LVDd, LVDs, AO, IVDd, LVPWD, LVEFI, LVFS, PAP
**Treatments & Outcomes**	12	Treatments: ACEI, BBs, Diuretics, IA (Inotropic Agents), Antibiotics, Hormones, IVIG<br>Outcomes: ROSSdis (ROSS at discharge), LVEFdis (LVEF at discharge), LOS (Length of Stay), Money (Cost), Death

Table S3. Core Variables Pre-Post Median imputation Sensitivity Analysis

Variable	Original Mean ± SD	Imputed Mean ± SD	Mean Change (%)	Median Change (%)	p-value
Demographics/Clinical					
Weight (kg)	15.30 ± 14.32	15.21 ± 14.24	-0.6	0	1
SBP (mmHg)	93.46 ± 17.68	93.15 ± 16.58	-0.32	0	0.9086
HR (bpm)	139.11 ± 38.65	139.02 ± 38.26	-0.06	0	0.9984
Cardiac Function					
LVEF (%)	51.47 ± 18.75	51.53 ± 18.53	0.12	0	0.986
LVDd (mm)	35.61 ± 15.29	35.47 ± 14.62	-0.39	0	0.0112
Biomarkers					
NT-proBNP (pg/mL)	10524.42 ± 10764.22	8384.71 ± 8386.23	-20.33	0	0
Cr (μmol/L)	38.57 ± 32.68	38.45 ± 32.46	-0.31	0	1
cTnI,ug/L	24.18 ± 497.22	14.83 ± 389.26	-38.66	0	0
ALT (U/L)	75.43 ± 261.09	75.21 ± 260.53	-0.29	0	1
AST (U/L)	115.31 ± 460.62	114.65 ± 458.52	-0.58	0	1
Comorbidities/Treatments					
Cardiomyopathy (%)	0.34 ± 0.47	0.34 ± 0.47	0	0	1
CHD (%)	0.37 ± 0.48	0.37 ± 0.48	0	0	1
IVIG Use (%)	0.30 ± 0.46	0.30 ± 0.46	0	0	1
BBs Use (%)	0.17 ± 0.37	0.17 ± 0.37	0	0	1
Malignant Arrhythmias (%)	0.06 ± 0.24	0.06 ± 0.23	-4.93	0	1

Supplementary data 3. Sensitivity Analysis of Median Imputation Impact on Core Variables. Original vs. imputed medians/means are shown for variables used in clustering. All binary variables (bottom section) had identical pre/post-imputation distributions (p=1).

Table S4. Top 20 Discriminating Variables Across Pediatric HF Phenotypes					
Variable	Cluster 0 (Median [IQR] or %)	Cluster 1 (Median [IQR] or %)	Cluster 2 (Median [IQR] or %)	P-value	Clinical Interpretation
BNP (pg/mL)	820.00 [211.50–2251.00]	466.50 [110–1938.25]	3234 [1039–5000]	<0.001	Cluster 2: Severe decompensation
NT-proBNP (pg/mL)	4293.00 [1179–10660]	3823.50 [1026.75–13389.75]	14448[5327.50–30000]	<0.001	Cluster 2: Worst prognosis
LVEF (%)	46.00 [32.00–60.00]	67.00 [60.00–73.00]	33.00 [26.00–43.00]	<0.001	Cluster 1: Preserved EF; Cluster 2: Severe systolic dysfunction
Cr (μmol/L)	45.80 [36.00–59.45]	25.00 [20.00–33.00]	28.20 [22.00–38.00]	<0.001	Cluster 0: Renal dysfunction
Age Group Distribution	98% Age Group 2-3	90.1% Age Group 0	Mixed Age Group 1	<0.001	Cluster 0: Older children; Cluster 1: Neonates
Cardiomyopathy (%)	43.90%	6.20%	49.90%	<0.001	Cluster 2: Acquired cardiomyopathy
CHD (%)	14.10%	72.20%	13.70%	<0.001	Cluster 1: Congenital dominant
Weight (kg)	30.00 [21.00–41.42]	5.50 [4.00–8.00]	7.95 [6.22–11.50]	<0.001	Cluster 0: Older/heavier
SBP (mmHg)	103.00 [94.00–112.00]	85.00 [78.00–94.00]	89.00 [81.00–97.00]	<0.001	Cluster 0: Hypertensive
HR (bpm)	106.00 [90.00–122.00]	145.00 [130.00–180.00]	140.00 [125.00–175.00]	<0.001	Cluster 1-2: Compensatory tachycardia
LVDd (mm)	47.00 [38.00–57.00]	24.00 [19.00–29.00]	39.00 [33.00–45.00]	<0.001	Cluster 0: Dilated cardiomyopathy
Preterm Birth (%)	11.40%	71.40%	17.10%	<0.001	Cluster 1: Prematurity-associated
ALT (U/L)	23.00 [14.55–41.00]	27.00 [18.00–43.35]	28.00 [18.00–56.28]	<0.001	Cluster 2: Hepatic congestion
AST (U/L)	35.00 [25.00–55.00]	44.90 [33.60–64.00]	49.00 [37.00–84.00]	<0.001	Cluster 2: Cardio hepatic syndrome
IVIG Use (%)	18.20%	35.30%	46.50%	<0.001	Cluster 2: Inflammatory activation
BBs Use (%)	54.50%	19.30%	26.20%	<0.001	Cluster 0: Beta-blocker responsive
cTnI (ug/L)	0.04 [0.01-0.19]	0.06 [0.02-0.23]	0.14 [0.03-0.48]	<0.001	Cluster 2:Acute catastrophic injury
Malignant Arrhythmias (%)	43.70%	22.20%	34.10%	<0.001	Cluster 0: Electrical instability
PAP (mmHg)	42.00 [30.00–61.00]	49.00 [34.00–70.00]	33.00 [22.00–45.50]	<0.001	Cluster 1: Pulmonary hypertension
Interrupted Feeding (%)	0.10%	65.30%	34.60%	<0.001	Cluster 1: Feeding intolerance

Values represent Median (IQR) (continuous variables) or percentage (categorical variables). Cluster 0: Chronic Hypertensive and Cardiorenal profile; Cluster 1: Preterm and CHD-Associated profile; Cluster 2: Fulminant Myocarditis and Cardiogenic Shock profile. Key clinical interpretations highlight phenotype-specific patterns (e.g., Cluster 2 shows highest BNP, lowest LVEF, and multiorgan injury markers).

Table S5. List of researchers (Arranged from high to low according to their contribution)

S.No.	Researchers along with their Affiliations
1	Children’s Hospital of Chongqing Medical University (Zhilin Huang, Huichao Sun, Lingjuan Liu, Tiewei Lv);
2	Henan Children's Hospital, Children's Hospital Afliated to Zhengzhou University (Fangjie Wang, Xiaochen Yan, Xiaoli Yao, Yingying Li);
3	Hunan Children's Hospital (Zhi Chen);
4	Shanghai Children’s Medical Center, School of Medicine, Shanghai Jiao Tong University (Ying Guo);
5	The Affiliated Hospital of Southwest Medical University (Xing Shen);
6	Children's Hospital of Fudan University (Fang Liu, Xuecun Liang, Yixiang Lin, Lan Ye);
7	Inner Mongolia People's Hospital (Hua Zhu, Yanyan Liang);
8	Shengjing Hospital of China Medical University (Yanlin Xing, Hong Wang, Wei Liu);
9	Fuwai hospital，Chinese Academy of Medical Sciences, Peking Union Medical College (Huili Zhang);
10	Hebei Children's Hospital (Yingqian Zhang, Bo Li);
11	Children's Hospital, Zhejiang University School of Medicine (Chunhong Xie, Yue Huang, Jianmei Zhu, Zhe Lin);
12	Children's Hospital of Nanjing Medical University (Shiwei Yang, Lianfu Ji);
13	Tianjin Children's Hospital (Shuhua Xing, Jiegang Deng);
14	The First Bethune Hospital of Jilin University (Yanyan Han, Shu Nie, Huaqing Sun);
15	The Second Hospital of Hebei Medical University (Lin Feng, Wei Xu, Haoyun Zhao);
16	The First Affiliated Hospital of Zhengzhou University (Jindou An, Song Feng);
17	Guangzhou Women and Children's Medical Center，Guangzhou Medical University (Li Zhang, Xiaofei Xie);
18	Qilu Hospital of Shandong University (Cuifen Zhao, Minmin Wang, Haizhao Zhao);
19	Qingdao Women and Children's Hospital (Zipu Li, Benzhen Wang, Guangsong Shan);
20	Xi'an Children's Hospital (Juanli Wang, Hongyu Xiao, Huan Li);
21	Tibet Autonomous Region People's Hospital (Bianbazhuoga, Mei Chen, Longya Qiao, Xiangyan Zhong, Yude Ma);
22	Jiangxi Provincial Children's Hospital (Junkai Duan, Fei Xu, Yunguo Zhou, Fang Xu);
23	Sichuan Academy of Medical Sciences & Sichuan Provincial People's Hospital (Qian Peng, Xiaoping Hu, Bo Li);
24	Guangdong Provincial People's Hospital, Guangdong Academy of Medical Sciences (Zhaofeng Xie, Yan Guan, Zhiwei Zhang, Shuishu Wang);
25	Anhui Provincial Hospital (Mei Xiong);
26	Xinhua Hospital Affiliated to Shanghai Jiaotong University School of Medicine (Yurong Wu);
27	Children's Hospital Affiliated to Shandong University, Jinan Children's Hospital (Lei Li, Xinxiang Li);
28	Beijing An Zhen Hospital of the Capital University of Medical Sciences (Yongmei Liang);
29	The First Affiliated Hospital of Shihezi University (Jinyong Pan, Qiang Gu, Fang Jiang);
30	Sichuan Provincial Maternity and Child Health Care Hospital (Xianmin Wang).
